# Serum lipoprotein(a), and plasminogen activator inhibitor-1 in uncomplicated type 2 diabetes mellitus: a case–control study

**DOI:** 10.1097/MS9.0000000000000915

**Published:** 2023-07-03

**Authors:** Fargeen E. Abdullah, Sardar N. Ahmed

**Affiliations:** Basic Science Department, College of Medicine, Hawler Medical University, Erbil, Iraq

**Keywords:** CAD, coronary artery disease, lipoprotein(a), metformin, plasminogen activator inhibitor-1, type 2 diabetes

## Abstract

Increased levels of lipoprotein (a) [Lp(a)] are indicative of coronary artery disease (CAD). Dyslipidemia, which is an established risk factor for CAD, is also an additional adverse effect of uncontrolled type 2 diabetes. Procoagulant and fibrinolytic indicators have been linked to the development of type 2 diabetes. Plasminogen activator inhibitor-1 (PAI-1), plays a significant negative regulatory role in fibrinolysis, through its function as the main inhibitor of tissue plasminogen activator. This study was designed to investigate the levels of Lp(a) and PAI-1 in uncomplicated type 2 diabetic patients and their association with healthy controls.

**Materials and methods:**

A case–control study designed for the estimation of Lp(a) and PAI-1 in serum after collecting blood samples from type 2 diabetic patients at the Layla Qasim Diabetic Center in Erbil, Iraq. The study included 90 participants, of whom 30 were healthy controls (15 males and 15 females). The remaining 60 cases were patients with type 2 diabetes with a duration of up to 6 years (30 males and 30 females).

**Results:**

Serum Lp(a) and PAI-1 levels were significantly lower in type 2 diabetic patients than in controls (*P*<0.01), this is an opposite result that usually happen in uncontrolled and complicated diabetic patients.

**Conclusions:**

The results were clearly stated a beneficial effect of Metformin on the levels of Lp(a) and PAI-1 in type 2 diabetes, so lowering their concentrations would help prevention of CAD, a known cause of death in diabetic patients.

## Introduction

Highlights

- Lower levels of serum lipoprotein (a) [Lp(a)] and Plasminogen activator inhibitor-1 (PAI-1) can be achieved in controlled and uncomplicated type 2 diabetes mellitus.
- The Metformin drug has a high impact on the levels of Lp(a) and PAI-1 in patients with type 2 diabetes; it significantly decreases their levels.
- The sex has no effect on the levels of serum Lp(a) and PAI-1.

Lipoprotein(a) [Lp(a)] is an LDL-like particle produced by the liver that contains an Apolipoprotein B-100 linked by a disulphide bridge to Apolipoprotein(a) [apo(a)]. The structure of the apo(a) component resembles that of plasminogen and competes with plasminogen for binding sites, which reduces fibrinolysis and increases the risk of blood clot formation^1,2^. Lp(a) is also thought to accelerate the development of atherosclerosis by binding LDL, calcium, and other substances into an atherosclerotic plaque on the blood vessel wall; this dual function of Lp(a) explains its role in the promotion of cardiovascular disease (CVD)^3^. Lp(a) is one of the most potent causative risk factors of atherosclerotic disease and is high in cholesteryl ester. Lp(a) is a marker for dyslipidemia and a stand-alone risk factor for cardiovascular events that has been associated with an elevated risk of myocardial infarction, stroke, and coronary heart disease^4^.

Plasminogen activator inhibitor-1 (PAI-1), a serine-protease inhibitor that is mostly produced by adipocytes, endothelial cells, and hepatocytes. PAI-1 is a key modulator of the fibrinolytic process and the higher levels of this antifibrinolytic protein are usually associated with insulin resistance and diabetes^5^. Diabetes is linked to a thrombotic environment due to increased platelet activation, prothrombotic coagulation factors, and impairment of the fibrinolytic system^6^. The fibrinolytic process is initiated by the conversion of plasminogen into plasmin, after being activated by tissue-type plasminogen activator or urokinase-type plasminogen activator. Plasmin is the primary protein that breaks down the fibrin fibers and produces fibrin breakdown products^7^. Instead of increased glucose levels, obesity and insulin resistance are the key factors contributing to elevated PAI-1 levels in diabetes. However, in the presence of insulin resistance, hyperglycemia may still have an impact. Protein accumulation at vascular atheroma sites, which seems to be particularly pronounced in people with diabetes, supports a function for PAI-1 in vascular disease^8^.

Diabetes mellitus is a group of metabolic diseases characterized by hyperglycemia caused by abnormalities in insulin secretion, insulin action, or both. Chronic hyperglycemia is linked to long-term harm, dysfunction, and failure of numerous organs’ normal functioning, including the eyes, kidneys, nerves, heart, and blood vessels^9,10^. However, diabetes is mostly classified into two major types: type 1 diabetes (IDDM) and type 2 diabetes (NIDDM). Type 2 diabetes (T2DM) and its complications constitute a significant global public health problem with high rates of morbidity and mortality^11^. Type 2 diabetes mellitus is a multifactorial metabolic condition marked by persistent hyperglycemia brought on by reduced insulin sensitivity or generation^12^. CVD is one of the consequences of chronic hyperglycemia; diabetic patients have a more than twofold increased risk of CVD-related death when compared to age-matched controls^13^. In both nondiabetic and diabetic individuals, Lp(a) is known to be a significant risk factor for atherosclerosis. In type 2 patients, as in nondiabetic individuals, in the majority of research involving NIDDM patients, Lp(a) is linked to atherosclerosis, and Lp(a) is a risk factor for CVD. However, different impacts of glycemic control on serum Lp(a). In type 2 diabetes mellitus, tightened glycemic control does not affect serum Lp(a)^4^. Biguanide is the first-line treatment for DM as it belongs to one of the main families of antidiabetic medications, among which metformin is the most widely used. Glucose management alone is insufficient; patients must also get counseling on the necessary lifestyle changes in addition to pharmaceutical therapies, such as oral antidiabetic medications.

The main goals of therapy are symptom improvement, mortality reduction, and risk reduction for the consequences of both microvascular and macrovascular diseases^14^. Metformin, in addition to its ability to decrease blood sugar, also improves lipid profiles, so it has cardiovascular preventive effects as a result of reducing risk factors for CVD such as obesity and dyslipidemia^15^. Agents that alter expression of PAI-1 in vivo is Metformin, which enhances circulating fibrinolytic activity. Early investigations with metformin showed an increase in overall fibrinolytic activity in those with diabetes, peripheral vascular disease, and coronary artery disease. Following administration of the insulin-sparing medication metformin, the rise of PAI-1 was significantly reduced^16^. Nagi *et al.* ^17^ found that metformin not only slows down the release of insulin but also slows the production of proinsulin and proinsulin split products, which can increase the expression of PAI-1. Nowadays, there is very little scientific information about the levels of serum Lp(a) and PAI-1 in type 2 diabetes around the world, and it is also an important fact that both of them combine in one role, which is that they prevent fibrinolysis process, a risk factor for CAD. This study is thus aimed to assess Lp(a) and PAI-1 levels in uncomplicated type 2 diabetic patients.

## Materials and methods

### Design of the study

A case–control study designed for the assessment of biochemical parameters (Lp(a) and PAI-1) in the serum of 90 participants. The participants were divided into two groups: case group (60 type 2 diabetic patients) were selected randomly with the duration of up to 6 years from those who were attending the diabetic center of Layla Qasim for there monthly routine medical examination in Erbil City, Iraq, and control group (30 healthy individuals). The present study was present based on strengthening the reporting of cohort studies in surgery (STROCSS) criteria^18^.

### Collection of blood samples

Blood samples were collected from both groups, (5–6) ml of blood samples was collected by venipuncture using sterile disposable syringe, then the serum was separated within an hour by centrifugation and refrigerated at −20^°^C for analysis.

### Method

The principles of estimation of serum Lp(a) and PAI-1 are based on the Sandwich-ELISA method. The kit’s Microelisa stripplate has an antibody precoating that is specific to [Lp(a) or PAI-1]. The appropriate Microelisa stripplate wells are filled with standards or samples, and the combination is introduced to the particular antibody. Then a Horseradish Peroxidase conjugated antibody specific for [Lp(a) in the case of Lp(a) estimation or PAI-1 in case of PAI-1 estimation] is injected into each Microelisa stripplate well, then incubated. Free parts are removed by washing. To each well, the TMB substrate solution is applied. Only those wells that contain [Lp(a) or PAI-1] and Horseradish Peroxidase conjugated [Lp(a) or PAI-1] antibodies will be blue in color and then changed to yellow after the addition of the stop solution. The optical density is measured spectrophotometrically at a wavelength of 450 nm. The optical density value is proportional to the concentration of [Lp(a) or PAI-1].

### Ethical approval

The Ethics Committee of Hawler Medical University granted ethical approval for the study (reference 8/2 on October 13, 2021). The written inform consent was obtained from participants.

### Statistical analysis

SPSS (Statistical Package for the Social Sciences) version 26 for Microsoft Windows 10 was used to conduct the statistical analysis. Normally distributed data were presented as mean ± SD. The independent sample *t*-test was done to compare the parameters between controls and cases. Statistical significance was considered to be (*P*<0.01).

## Results

Table 1 displays the demographic details of the study groups, including the number, (Mean±SD) of ages and BMI for the case and control groups. There were no significant differences in ages and BMI between groups (*P*>0.01). The mean levels of serum Lp(a) was significantly lower in the case group when compared to the control group as shown in Table 2 with *P*-value (<0.01), while there were no significant differences in the mean levels serum Lp(a) between males and females of case group (*P*>0.01). The mean levels of serum PAI-1 in Table 3 showed a significant lower in case group than in the control group with a *P*-value (<0.01), while there were no significant differences in the mean levels of serum PAI-1 between males and females of case group (*P*>0.01).

**Table 1:** Sex, number, age, and BMI of case and control groups

Parameters	Sex	Case No.	Mean ± SD	Range	Control No.	Mean±SD	Range
Age (years)	Males	30	51.03±8.23	(40–70)	15	50±7.57	(41–70)
	Females	30	52.20±7.73	(40–66)	15	46.66±5.51	(40–56)
	Both	60	51.61±7.94	(40–70)	30	48.33±6.72	(40–70)
BMI (Kg/m^2^)	Males	30	29.58±4.84	(22.72–43.34)	15	28.41±5.16	(19.31–38.28)
	Females	30	30.59±4.31	(22.60–39.19)	15	30.23±2.66	(26.04–33.53)
	Both	60	30.08±4.57	(22.60–43.34)	30	29.32±4.14	(19.31–38.28)

**Table 2:** Serum lipoprotein(a) levels in ng/ml in case and control groups with test of comparison

Variables	Case No.	Mean ± SD	Range	Control No.	Mean ± SD	Range	*P*
Males	30	92.95±12.73	(69.87–118.26)	15	124.25±10.98	(107.29–146)	*P*<0.01
Females	30	92.84±11.03	(73.10–114.39)	15	118.27±10.55	(98.58–138.90)	*P*<0.01
Both (Male and Female)	60	92.90±11.81	(69.87–118.26)	30	121.26±11.01	(98.58–146)	*P*<0.01

**Table 3:** Serum plasminogen activator inhibitor-1 levels in ng/ml in case and control groups with test of comparison

Sex	Case No.	Mean ± SD	Range	Control No.	Mean ± SD	Range	*P*
Males	30	33.88±9.25	(19.12–52.21)	15	46.62±6.74	(34.26–58.38)	*P*<0.01
Females	30	33.77±8.16	(18.82–46.62)	15	45.67±7.88	(35.74–64.56)	*P*<0.01
Both	60	33.83±8.65	(18.82–52.21)	30	46.15±7.22	(34.26–64.56)	*P*<0.01

## Discussion

In this case–control study, our findings revealed that there were a significantly lower levels of serum Lp(a) among the type 2 DM patients when compared to control healthy individuals. This is an opposite result of many researches, in which their results showed an increase in the levels of Lp(a) in type 2 diabetic patients such as in^4,19,20^. So, to know why we got these results, we thought more and more and focused on the questionnaires that we filled by the patients. As a result, we achieved many important facts which leaded to such results. First of all, our patients are newly diagnosed with type 2 DM the maximum duration was 6 years, for that most of patients controlled their blood glucose by diet; exercise; taking medication mostly Metformin drug; monitoring their blood glucose by using Glucometer device in the home and with measuring their HbA1c level every 3 or 6 months in the diabetic center; and all of them were free from diabetic complications. So in our study, from 60 diabetic patients 53 of them were used Metformin drug as a treatment, which is equal to 88% from all patients and this is a high percentage that must be taken in consideration. This present study showed a very clear impact of Metformin on the level of Lp(a), by getting a lower level of Lp(a) in type 2 diabetic patients in compare to control healthy individuals, and this result is similar to the results of many researches like^21,22^. Metformin is a common oral hypoglycemic medication for type 2 diabetes mellitus. Metformin is frequently used to treat diabetes, because it does not increase the process of insulin secretion and cannot result in a blood glucose deficit. Metformin can also cause weight loss in a person^23^. Metformin stimulates the activation of adenosine monophospho protein kinase inhibiting the anabolic route and promoting the catabolic pathway. Adenosine monophospho protein kinase, which is found in the heart, has the ability to suppress sterol regulatory element-binding protein 1, a protein that regulates the genes required for the lipogenesis process. As a result of this downregulation, fatty acid desaturase is activated, which lowers arachidonic acid levels. This reduction may result in an increase in membrane fluidity, which helps to preserve the integrity and function of the cell membrane, thereby increasing the recycling of LDL-C receptors and lowering LDL-C levels^24^. Several studies found that metformin monotherapy significantly lowered LDL levels. This is demonstrated by Lin *et al.*’s study^25^, which found that Metformin can lower LDL levels in those with type 2 diabetes mellitus. Since Lp(a) is a modified form of LDL (in which Apo-A-1 is linked to Apo-B) and is physiologically separate from LDL, its levels are genetically set. An increased risk of CVD has been associated with higher plasma levels of Lp(a) and LDL cholesterol. Lp(a) is a dyslipidemic marker and independent risk factor for cardiovascular incidents, correlated with an increased risk of myocardial infarction, stroke, and coronary heart disease^21^. A decrease in insulin resistance by Metformin treatment may reduce the [Lp(a)] levels. Metformin may work to reduce insulin resistance by preventing the hepatic synthesis of glucose via gluconeogenesis by direct inhibition of gluconeogenic enzymes, reduce the absorption of intestinal glucose and raise the sensitivity of peripheral organs to insulin^26^. However, the mechanism of Metformin’s effects on [Lp(a)] are unknown.

The same effect of Metformin on the levels of PAI-1 showed by numerous studies, in which findings have demonstrated that Metformin reduces the blood levels of PAI-1 in persons with diabetes and other insulin-resistant diseases^16^. Recent investigations employing tests specific for the fibrinolytic system’s components have demonstrated that Metformin’s effects result in a decrease in plasma levels of the fibrinolytic inhibitor, PAI-1^27^. The link between vascular disease and reduced fibrinolysis may be brought on by high levels of PAI-1 according to studies, and evidence to support the idea that decreasing PAI-1 would be advantageous in this respect.

## Conclusions

The study revealed that lower levels of serum Lp(a) and PAI-1 can be achieved in controlled and uncomplicated type 2 diabetes mellitus, so lowering their levels would help prevent CAD. Moreover, the Metformin drug has a high impact on the levels of Lp(a) and PAI-1 in patients with type 2 diabetes; it significantly decreases their levels. Finally, sex has no effect on the levels of serum Lp(a) and PAI-1.

## Ethical approval

The Ethics Committee of Hawler Medical University College of Medicine granted ethical approval for the study (reference 8/2 on October 13, 2021).

## Consent

The written inform consent was obtained from participants.

## Funding

None.

## Author contribution

All authors made a significant contribution in the conception, study design, execution, acquisition of data, analysis, and interpretation; took part in drafting, revising, or critically for important intellectual content; gave final approval of the version to be published; have agreed on the journal to which the article has been submitted; and agree to be accountable for all aspects of the work.

## Conflicts of interest disclosure

The authors declare that they have no competing interests.

## Research registration unique identifying number (UIN)

1. Name of the registry: NA.
2. Unique Identifying number or registration ID: NA.
3. Hyperlink to your specific registration (must be publicly accessible and will be checked): NA.

## Guarantor

Fargeen E. Abdullah.

## Data availability statement

All the data of this study are available from the corresponding author upon reasonable request.

## Provenance and peer review

Not commissioned, externally peer-reviewed.

## Acknowledgements

The authors express their heartfelt thanks to all individuals who participated in the study.
